# Primary Human Hepatocytes, But not HepG2 or Balb/c 3T3 Cells, Efficiently Metabolize Salinomycin and Are Resistant to Its Cytotoxicity

**DOI:** 10.3390/molecules25051174

**Published:** 2020-03-05

**Authors:** Lidia Radko, Małgorzata Olejnik, Andrzej Posyniak

**Affiliations:** Department of Pharmacology and Toxicology, National Veterinary Research Institute, 57 Partyzantow Avenue, 24-100 Pulawy, Poland; lidia.radko@piwet.pulawy.pl (L.R.); aposyn@piwet.pulawy.pl (A.P.)

**Keywords:** salinomycin, cytotoxicity, metabolites, interaction, human, in vitro

## Abstract

Salinomycin is a polyether antibiotic showing anticancer activity. There are many reports of its toxicity to animals but little is known about the potential adverse effects in humans. The action of the drug may be connected to its metabolism. That is why we investigated the cytotoxicity of salinomycin and pathways of its biotransformation using human primary hepatocytes, human hepatoma cells (HepG2), and the mouse fibroblast cell line (Balb/c 3T3). The cytotoxicity of salinomycin was time-dependent, concentration-dependent, and cell-dependent with primary hepatocytes being the most resistant. Among the studied models, primary hepatocytes were the only ones to efficiently metabolize salinomycin but even they were saturated at higher concentrations. The main route of biotransformation was monooxygenation leading to the formation of monohydroxysalinomycin, dihydroxysalinomycin, and trihydroxysalinomycin. Tiamulin, which is a known inhibitor of CYP450 izoenzymes, synergistically induced cytotoxicity of salinomycin in all cell types, including non-metabolising fibroblasts. Therefore, the pharmacokinetic interaction cannot fully explain tiamulin impact on salinomycin toxicity.

## 1. Introduction

With the increasing role of cancer in public health, the search for new drugs becomes even more important. For this purpose, huge screening tests of already known chemicals are among the applied strategies. Using this approach, scientists have found several candidates for anticancer drugs among polyether ionophores. One of them is salinomycin (SAL)—a compound used for nearly 50 years as an anti-parasitic in veterinary practice. Gupta et al. demonstrated that SAL selectively targeted breast cancer stem cells [1]. The drug shows anti-tumorigenic properties in various types of cancer. It sensitizes multidrug resistant cancer cells to other chemotherapeutic agents [2]. According to many researchers, SAL is one of the biggest hopes in oncology [1,3,4].

At the same time, the potentially toxic side effects of the drug are a major concern. So far, little is known about the toxicity of SAL to humans. An accidental poisoning of a feed mill worker with SAL revealed severe toxicity with nausea, photophobia, limb weakness, tachycardia, muscle pain, and mild rhabdomyolysis with myoglobinuria [5]. According to the risk assessment published by the European Food Safety Authority, the acceptable daily intake (ADI) of SAL is 5 µg/kg b.w. based on the NOAEL (No Observed Adverse Effect Level) of 500 µg/kg b.w, derived from chronic toxicity study in dogs [6].

The animal experiments and field cases of poisonings have shown that the toxicity of SAL is highly dependent on animal species. Turkeys and horses are the most sensitive among the reported species with the LD_50_ of 0.6 mg/kg b.w., in comparison to LD_50_ 50 mg/kg b.w. and 21 mg/kg b.w. in rats and rabbits, respectively [7]. The low therapeutic index of SAL and high susceptibility of certain species to its toxic properties have led to poisonings of both target and non-target husbandry animals [7,8]. The species-dependent toxicity is not fully understood but it seems to be connected with the metabolism of the compound.

Some simultaneously administered drugs, e.g., tiamulin or chloramphenicol increase SAL toxicity [7,8]. This effect is generally connected with their influence on drug-metabolizing enzymes. Tiamulin shows the most prominent clinical effects of interaction with SAL and is a known inhibitor of CYP3A enzymes, which suggests that it interferes with SAL pharmacokinetics. The in vitro experiments on biotransformation of monensin in different animal species reinforce this conclusion [9]. On the other side, glucocorticoids induce CYP3A4 izoenzymes hence influencing ionophores metabolism [9,10].

However, our previous research performed using rat cell models are not fully in line with this hypothesis. Although tiamulin synergistically increased the cytotoxicity of SAL and affected its biotransformation, the strength of the pharmacokinetic interaction only partially explained the pharmacodynamic effect [11]. What is especially interesting, tiamulin influenced the viability of metabolically inactive rat myoblasts showing zero biotransformation of SAL. Further studies involving qualitative and quantitative analysis of the drug metabolites are, therefore, necessary to ultimately assess their role in SAL toxicity.

Taking into account the mentioned interspecies differences, the target organism should be used for toxicological evaluation. The use of cell models is, therefore, an indispensable tool for studying SAL toxicity in humans. Primary human hepatocytes (PHH) have been extensively used for testing drugs at both a pre-clinical and clinical stages because of the special role of the liver in the metabolism of drugs and organism detoxification in vivo. Human hepatoma (HepG2) cells were selected as target cells for evaluating toxicity for human liver cancer and have been extensively used as the test system for the prediction of toxicity and metabolites in cancer patients [12]. Balb/c 3T3 fibroblasts are commonly used for the general toxicity assessment and serve as a reference non-metabolizing model [13].

The aim of the study is to assess the cytotoxicity and metabolism of SAL as well as its interactions with tiamulin or prednisolone. Hepatocytes were used to mimic optimal metabolism in a healthy organism. Hepatoma can serve as an indicator of the cytotoxic potential and metabolism of SAL in cancer cells, and fibroblasts represent non-metabolizing cells. The use of these three different cell models provides complementary data to significantly increase knowledge on pharmacological and toxicological properties of SAL.

## 2. Results

### 2.1. Cytotoxicity of Salinomycin and its Combinations with Tiamulin or Prednisolone

The viability of primary human hepatocytes (PHH), human hepatoma (HepG2) cells, and fibroblasts (Balb/c 3T3) after exposure to SAL alone or in co-action with tiamulin or prednisolone was time-dependent, concentration-dependent, and assay-dependent (Supplementary Material, Figures S1, S2, S3). The viability of cells co-treated with tiamulin was decreased compared to SAL alone. The addition of prednisolone slightly modulated cells viability in both directions, depending on the model and endpoint.

In PHH, SAL first affected lysosomal activity (measured by neutral red uptake test, NRU) at the concentration of 1.56 µg/mL after 24 h (Supplementary Material, Figure S1) [13]. After this time, in HepG2 and Balb/c 3T3 cell lines, the significant effect was noted already at 0.39 µg/mL of SAL alone or in both drug combinations in two endpoints: lysosomal activity and total protein content (TPC) (Supplementary Material, Figures S2 and S3) [14].

In human hepatocytes, the EC_50_ values (effective concentration) for SAL alone or its combination with tiamulin or prednisolone were calculated in NRU and the lactate dehydrogenase (LDH) assay [15]. For two other assays, they exceeded tested concentration range. The EC_50_ values for SAL co-action with tiamulin were significantly lower than for SAL alone. In the case of human hepatocytes co-treated with prednisolone, the EC_50_ values were significantly changed in NRU but not in the LDH assay when compared to the results for SAL alone (Table 1).

The lowest EC_50_ values for SAL alone and its combination with tiamulin or prednisolone were obtained for HepG2 cells in the NRU assay. The values obtained in other tests were much higher (>3 µg/mL). The EC_50_ values for SAL co-action with 10 µg/mL of tiamulin was significantly lower in all used assays when compared to the results for SAL alone or in combination with 1 µg/mL of tiamulin. The addition of prednisolone significantly decreased EC_50_ values in TPC (48 h) and LDH (48 h) assays when compared to the values for SAL alone (Table 1).

The lowest EC_50_ values for SAL and its combination with tiamulin or prednisolone were obtained for the fibroblasts in the NRU assay. These values significantly decreased for SAL co-exposure with 10 µg/mL tiamulin in MTT [16] and TPC when compared to the values for SAL alone or its co-action with 1 µg/mL tiamulin. In fibroblasts co-treated SAL with prednisolone for 24 h, the EC_50_ values statistically increased in TPC and MTT assays when compared to SAL alone, but this effect has receded (TPC) or even reversed (MTT) after 48 h of exposure (Table 1).

The EC_50_ results obtained in PHH after 24 h exposure were 30 times higher in the NRU assay when compared to the values in HepG2. The EC_50_ values of SAL after 24 h exposure of PHH and HepG2 cells were lower in the LDH assay but higher in the MTT, NRU, and TPC assay to the results obtained for Balb/c 3T3 fibroblasts (Table 1).

**Figure 1:**
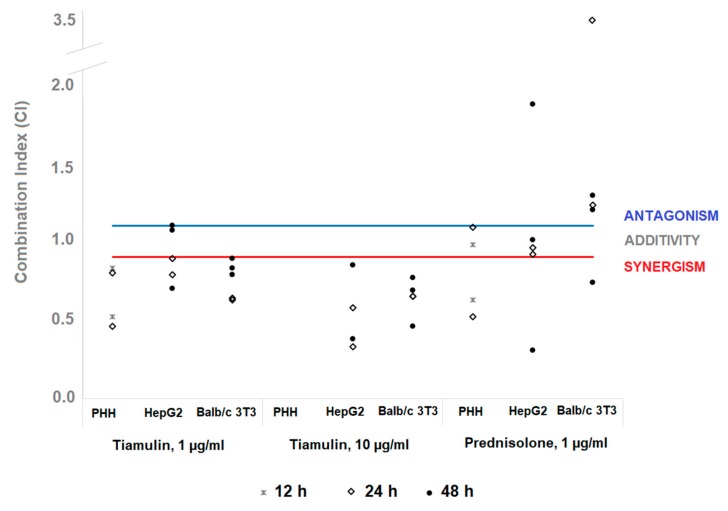
The combination index (CI) used for the denomination of the mode of interaction between salinomycin and tiamulin or prednisolone. The cut-off values for synergism and antagonism (0.9 and 1.1, respectively) are shown for comparison. CI was assessed for three cell models in three exposure times, each during three independent biological experiments. Every dot represents a value calculated with a specific endpoint (MTT, NRU, TPC, and LDH assay). The calculation of CI was not possible for all combinations of the model, exposure time, and endpoint.

The combination index analysis was adopted to determine the type of interaction between SAL and tiamulin or prednisolone on PHH, HepG2 cells, and fibroblasts (Balb/c 3T3), as shown in Figure 1. The interaction between SAL and tiamulin in human hepatocytes showed a considerable synergistic (CI = 0.5–0.8) mode. The interaction between the ionophore and prednisolone showed a synergistic mode (CI = 0.5–0.6) in the NRU assay but additive effects in the LDH assay.

The mode of interaction between SAL and tiamulin at concentration 1 µg/mL in HepG2 cells displayed a synergistic (CI = 0.7–0.9) action in MTT (24 h) and TPC assays. However, weak antagonistic (CI = 1.1) effects were observed in the MTT and LDH assay after 48 h of exposure. The strong synergistic (CI = 0.3–0.8) mode of interaction was observed between the ionophore and tiamulin at a high concentration (10 µg/mL) in all assays. Prednisolone acted as a SAL antagonist in the MTT assay. It showed a synergistic or additive mode of interaction in TPC and LDH depending on the time of exposure (Figure 1).

The mode of interaction between SAL and tiamulin at both concentrations displayed synergistic effects (CI = 0.5–0.9) in Balb/c 3T3 cells. The antagonistic effect (CI= 1.2–3.3) of SAL co-action with prednisolone was observed. However, synergistic action in the MTT (48 h) assay was reported (Figure 1).

To sum up, tiamulin increased synergistically cytotoxicity of SAL in all cell models and assays except for single results in HepG2 cells after 48 h of exposure. The CI values for SAL-tiamulin interaction decreased with the increasing concentration of tiamulin and increased with the time of incubation. For the interaction with prednisolone, no consistent pattern was visible in human liver cell models. In fibroblasts, most tests indicated that prednisolone acted as a SAL antagonist.

### 2.2. Metabolism of SAL

**Figure 2:**
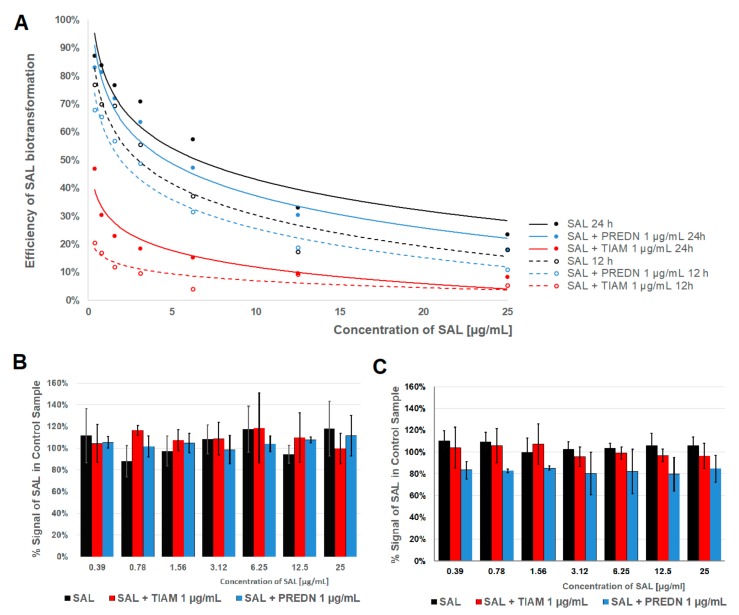
Efficiency of biotransformation of salinomycin (SAL) in three cell models. (**A**) Percent decrease of the SAL signal in comparison to control samples in PHH exposed to drug(s) for 12 and 24 h. (**B**) Percent of the SAL signal in comparison to control samples in Balb/c 3T3 fibroblasts exposed to drug(s) for 24 h. (**C**) Percent of the SAL signal in comparison to control samples in HepG2 cells exposed to drug(s) for 24 h.

SAL was effectively metabolised only by PHH (Figure 2A). Fibroblasts expressed no biotransformation (Figure 2B) and HepG2 cells metabolised ca. 20% of SAL only after their induction with prednisolone (Figure 2C). The percentage of SAL metabolised by PHH decreased with the increasing concentration of the ionophore added to cell medium and increased with the time of exposure. Addition of prednisolone slightly decreased the extent of SAL metabolism both after 12 and 24 h, but this effect was not statistically significant. The maximum percentage of bio-transformed SAL reached only 25% in cells co-exposed with tiamulin.

The signals differentiating the tested samples and controls included the following precursor ions: *m*/*z* 760.1 (SAL − 14 Da), *m*/*z* 776.1 (SAL + 2 Da), *m*/*z* 790.1 (SAL + 16 Da), *m*/*z* 806.1 (SAL + 32 Da), and *m*/*z* 822.1 (SAL + 48 Da). Twenty peaks were at least twice as high as in the controls representing abiotic degradation and they were assigned as potential SAL metabolites (Table 2, Supplementary material Figure S4).

The main route of metabolism of SAL in human hepatocytes was monooxygenation leading to the formation of monohydroxysalinomycin (*n* = 3), dihydroxysalinomycin (*n* = 14), and trihydroxysalinomycin (*n* = 1). Additionally, three demethylated compounds of low abundance were detected. The four most abundant metabolites, constituting almost 90% of the total signal were two isomers of hydroxy-salinomycin (M12 and M14) and two of the di-hydroxylated derivatives (M2 and M6). Two of the most abundant metabolites were formed in HepG2. Additionally, M2 and M6 compounds appeared after exposure of hepatoma cells to a combination of SAL and prednisolone (Table 2).

As seen in Table 2, prednisolone did not disturb the proportions among the metabolites produced by PHH. In addition, in the function of time and concentration of SAL, the proportions among the metabolites changed only slightly. In the cells exposed to tiamulin, mono-hydroxylated SAL metabolites (M12, M14) were more prominent in comparison to SAL and SAL-prednisolone treated cells and the contribution of more efficiently metabolized derivatives was reduced. The production of the most hydrophilic compounds (M1, M2, M3, M4) was decreased about two times.

## 3. Discussion

SAL has been demonstrated to effectively target cancer, including multidrug and apoptosis-resistant cells [1]. Since this discovery in 2009, many research papers were published focusing on the potential application of this drug in oncology. So far, SAL was proven to be efficient in vitro against, e.g., breast, liver, and colorectal cancer [2,3,17,18,19,20]. Although the cytotoxicity of SAL against normal cells was significantly lower in comparison to cancer ones, there are still some knowledge gaps concerning the safety of this drug [21]. Little is known about its pharmacokinetics.

In the present study, in order to learn the toxicity of SAL and identify its metabolites, human hepatocytes and hepatoma (HepG2) cells were used since these are stable and robust in vitro models used to study the metabolism of drugs. The fibroblasts (Balb/c 3T3) were compared above to determine the role of metabolism of SAL in its toxicity.

The battery of tests estimating different endpoints were performed. The lysosomal activity was the most strongly inhibited, which is in accordance with our previous study using rat cellular models [11]. Yet, NRU is not a definitive test of lysosomal activity. The observed inhibitory effect of SAL is in line with its inhibitory effect on autophagy and anticancer activity [4]. This mechanism of action is associated with the impact of SAL on cellular ion homeostasis by suppressing autophagy flux and lysosomal proteolytic activity [4,22]. The formation of lipid-soluble complexes with the monovalent cations (especially Na^+^ and K^+^) disturbs their transport through cell membranes. It leads to the increase of intracellular Ca^2+^ levels, which is followed by decreased energy and inhibits lysosomal protein degradation by affecting pH inside the organelle [2,11,17,23].

SAL is found to both induce and inhibit autophagy [4]. The contrasting reports on the effects of SAL on autophagy may reflect the time after exposure and different concentrations. Longer exposure and higher concentrations usually lead to autophagy inhibition, which is often observed with other natural compounds [24].

The toxicity of SAL in PHH is very unique. In our study, SAL exerted toxic effects on PHH at higher concentrations (>10 µg/mL), but at lower concentrations, it was well tolerated. Similarly, in the study of Klose et al., SAL impaired cell functions only when administered in higher concentrations (5–10 µM, 3.76–7.51 µg/mL). Cells completely recovered within five days after ceasing the exposure and no apoptosis was observed [25]. Wang et al. showed that, in their in vivo studies on mice, SAL at doses of 4 and 8 mg/kg b.w. did not induce apoptosis in normal liver cells, while, at the same time, significantly reducing the hepatoma tumor [18]. All these observations are in line with our findings and confirm low toxicity of SAL to normal liver cells.

The cytotoxicity of SAL to cell lines highly depends on the model, assay, and exposure conditions. The EC_50_ values varied from 0.1 µM (0.08 µg/mL) to 30 µM (22.5 μg/mL) [1,11,19,26,27]. In our study, cytotoxic concentrations of SAL for human hepatoma cells were in the higher end of the cited concentrations’ range and reached 0.8–20.5 µM (0.6–15.4 µg/mL) after 24 h [18,25,26]. The HepG2 cells were more sensitive to SAL toxicity than human hepatocytes. Likewise, human (HepG2) and chicken (LMH) hepatoma lines were more sensitive to SAL cytotoxicity than normal rat myoblasts (L6) [26].

SAL effectively kills both cancer stem cells (CSCs) and multidrug-resistant cancer cells (MDR) [1,3]. Recent studies have demonstrated that SAL inhibits growth, migration, and invasive properties of hepatocellular carcinoma cells [20]. The ability of the drug to induce massive apoptosis in HepG2 cells is potentially based on the inhibition of Wnt/β-catenin signaling via increased intracellular Ca^2+^ levels [18]. Another proposed mechanism is the dysfunction of mitochondria with increased ROS production, which are associated with suppression of autophagy and induction of apoptosis in HepG2 cells but not in PHH [4,25].

Fibroblasts were more sensitive to SAL than PHH or HepG2 cells. The cytotoxic concentrations ranged from 0.6 to 10 µg/mL for inhibiting lysosomal and mitochondrial activity, and synthesis of cellular proteins. However, the concentration disrupting the cellular membrane was higher than for hepatic cells. Regarding the concept that SAL may target highly proliferative cells as opposed to other cells, more slowly growing cells are supported by our data showing that the drug is toxic to fibroblasts at levels lower than for liver cells [18].

PHH were the most resistant to SAL cytotoxicity among the tested models. They were also the only cells efficiently metabolizing SAL, up to 90% of the dose, which is greater than what rat hepatocytes could do [11]. In the in vivo studies, the metabolism of SAL in mice, rat, and chicken was highly efficient with unchanged SAL in excreta representing only 2% of the administered dose [28]. In contrast to normal liver cells, HepG2 hepatoma presented low potency to metabolize SAL, which is comparable to the rat hepatoma (FaO) in our previous study [11,29]. These results confirm again that hepatoma-derived cells lack metabolic activity of CYP enzymes [30]. As expected, fibroblasts did not bio-transform the drug at all.

The detected metabolites were compatible with our previous findings and other reports [11,28,31]. SAL was hydroxylated with up to three oxygen atoms connected to the molecule in comparison to pentahydroxy-salinomycin found during in vivo studies in murine and chicken [28]. The reason for this difference in the extent of SAL biotransformation can be two-fold. First, even primary hepatocytes lose some of the normal liver metabolic activity when cultured in the mono-layer [30]. Second, inter-species variation may play a role. However, for monensin (a related compound), this factor influenced only the ratio among metabolites and not the formation of individual derivatives [32].

Of the four main metabolites (representing above 2% of the total signal), three compounds (M2, M12, and M14) were produced by rat hepatocytes and detected as highly abundant [11]. One dihydroxy- metabolite (M6) was, however, not produced in rat cell cultures. Vice versa, two SAL trihydroxy-derivatives formed in rat hepatocytes were not found in this study. Besides these differences, the metabolites were identical, but their relative abundance differed significantly.

Simultaneous administration of tiamulin and ionophore antibiotics has led to the toxic interaction in animals, which even caused death [8]. Similarly to our previous study, we confirmed that SAL was more cytotoxic in combination with tiamulin [11]. The synergistic interaction between tiamulin and SAL was confirmed by a combination index (below 1), correlated with tiamulin concentration.

Monoxygenation as the main route of metabolism of SAL confirms that CYP450 inhibitors can influence the pharmacokinetics of SAL. That was suggested before as the mechanism for interaction of ionophores with tiamulin, which increased toxic properties of these drugs [9,28,32]. In our study, tiamulin inhibited metabolism of SAL in PHH by reducing the overall biotransformation and shifting it to less oxidised metabolites. However, the synergistic toxicity of SAL with tiamulin cannot be fully explained by the pharmacokinetic interaction. The interaction was observed in non-metabolizing cell models (rat myoblasts and mouse fibroblasts) and poor-metabolising rats and human hepatoma [11].

On the other side, glucocorticoids induce CYP3A4 izoenzymes, which has been proven to affect metabolism of another ionophore, monensin [9,10]. The co-exposure to prednisolone, however, did not have the clear impact on the cytotoxicity and the metabolism of SAL. The decreased cytotoxicity of SAL was observed in fibroblasts and with some endpoints in hepatoma. In contrast, SAL and prednisolone showed either synergistic or an additive action in PHH. The efficiency of biotransformation was increased in HepG2 cells but not in normal hepatocytes showing the exact same pattern as rat liver cells [11]. The time of exposure might have played a role even though Pascussi et al. observed increased expression of CYP3A4 mRNA already after 24 h of incubation with dexamethasone [33]. Again, like for tiamulin, the pharmacokinetic interaction does not fully explain the impact on toxicity.

The results confirm that SAL acts as an anticancer drug. The toxicity of this compound depends on its metabolism as the efficient SAL biotransformation reduced its toxicity. However, it is not the only factor influencing cells susceptibility, as shown in the study of interaction with tiamulin using non-metabolizing or poor-metabolizing cells. Therefore, further studies are needed to fully understand the mechanism of species’ sensitivity to SAL.

## 4. Materials and Methods

### 4.1. Chemicals and Reagents

Analytical standards of SAL monosodium salt hydrate (SAL, CAS: 55721-31-8), tiamulin (T, CAS: 55297-96-6), prednisolone (P, CAS: 50-24-8), and monensin sodium (MON, CAS: 22373-78-0) were purchased from Sigma–Aldrich Co. (St. Louis, MO, USA). Triton X-100, trypan blue, dimethyl sulfoxide (DMSO), fetal bovine serum (FBS), bovine calf serum (BCS), neutral red dye (NR), coomassie brilliant blue R-250 dye, 3-(4,5-dimethylthiazol-2-yl)-2,5-diphenyltetrazolium bromide (MTT), trypsin-EDTA, and antibiotic solution (10,000 U/mL of penicillin, 10 mg/mL of streptomycin) were purchased from Sigma–Aldrich Co. (St. Louis, MO, USA). Acetonitrile, methanol, ammonium formate, formic acid, all HPLC, or LC-MS grade were purchased from J.T. Baker (Phillipsburg, NJ, USA). All other chemicals were purchased from commercial suppliers and were of the highest available purity.

### 4.2. Cell Cultures

#### 4.2.1. PHH and Culture Conditions

Cryopreserved PHH were purchased from the Primacyt GmbH (Schwerin, Germany). Two different lots were chosen based on CYP activity. The donor was a 59-year-old female. The post-thaw viability was 94.6% for lot HH160531 (used in two repetitions) and 85.0% for lot HH130805. The initial cell viability was determined with the trypan blue exclusion test. The cells were thawed and purified using the Plating and Thawing Kit (Primacyt GmbH). Purified hepatocytes were resuspended in Hepatocyte Plating Medium (Primacyt GmbH) and seeded on collagen-coated 96-well plates at a density of 9 × 10^5^ cell/mL and incubated at 37 °C with 5% CO_2_. After 6 h of incubation, the medium was removed and the cells were washed with PBS. Then Human Hepatocyte Maintenance Medium (HHMM) (Primacyt GmbH) with the studied drugs was added to hepatocytes (100 µL/well). The culture conditions were kept according to the manufacturer’s protocol [34].

#### 4.2.2. Cell lines and Culture Conditions

The HepG2 cell line was purchased from the American Type Culture Collection (ATCC^®^ HB-8065™). The cells were cultured in Minimum Essential Medium Eagle (MEME) (Sigma–Aldrich Co. (St. Louis, MO, USA)). Normal murine embryonic fibroblasts, Balb/c 3T3 clone A31 cell line (gift from Department of Swine Diseases of the National Veterinary Research Institute in Pulawy) was cultured in Dulbecco’s Modified Eagle’s Medium (DMEM) (Invitrogen Corporations (Paisley, UK)). The media were supplemented with 10% BCS (Balb/c 3T3), 10% FBS (HepG2), 1% L-glutamine, and 1% antibiotic solution. The cells were maintained in 75 cm^2^ cell culture flasks in humidified incubator at 37 °C, in an atmosphere of 5% CO_2_. The medium was refreshed every two or three days and the cells were trypsinized by 0.25% trypsin–0.02% EDTA after reaching 70–80% confluence. The initial cell viability was determined with the trypan blue exclusion test. Single cell suspensions were prepared and adjusted to a density of 2 × 10^5^ cell/mL (HepG2) and 1 × 10^5^ cell/mL (Balb/c 3T3). The cell suspension was transferred to 96-well plates (100 µL/well) and incubated for 24 h before exposure to the studied drugs.

### 4.3. Exposure to Drugs

The concentration ranges of the drugs were chosen according to their solubility and their plasma level. Each drug was dissolved in DMSO. The final concentration of DMSO was 0.1% or 0.2% in the medium for both samples and corresponding controls. The medium used for test solutions and in control preparation did not contain serum and antibiotics. All drug solutions in medium were freshly prepared and protected from light. SAL was tested in seven concentrations from 0.39 to 25 µg/mL. Tiamulin was added in two concentrations (1 and 10 µg/mL) or just one concentration (1 µg/mL) to cell lines or human hepatocytes, respectively. Prednisolone was tested in one concentration—1 µg/mL independent of the type of cells. Each concentration of SAL or the combination of the ionophore with tiamulin or prednisolone were tested in six replicates during three independent experiments. The cytotoxicity was assessed after 24 and 48 h of exposure of cell lines or after 12 and 24 h exposure of PHH. The medium was not changed during the incubation time.

### 4.4. Cytotoxicity Assessment

#### 4.4.1. MTT Assay

The metabolic activity of living cells was assessed by measuring the activity of dehydrogenases [16]. After incubation of the cells with the drug(s), 10 μL of the 3-(4,5-dimethylthiazol-2-yl)-2,5-diphenyltetrazolium bromide (MTT) solution (5 mg/mL in PBS) was added to each well of 96-well plates and incubated. After 3 h, the MTT solution was removed and the intracellular formazan crystals were dissolved in 100 µL of DMSO. The plate was shaken for 15 min at room temperature and transferred to a microplate reader to measure the absorbance at 570 nm, using a blank as a reference. Cytotoxicity was expressed as a percentage of the negative control (0.1% or 0.2% DMSO).

#### 4.4.2. NRU Assay

The assay, based on staining of living cells by neutral red (NR), was performed according to the protocol described by Borenfreund and Puerner [13]. After the incubation, the medium containing the drug was removed and the cells were washed with PBS. Then 100 µL/well of NR solution (50 µg/mL) was added for 3 h. After this time, the cells were washed with PBS. The dye from viable cells was released by extraction with a mixture of acetic acid, ethanol, and water (1:50:49, *v*:*v*:*v*). After 10 min of shaking, the absorbance of the dissolved NR was measured at 540 nm using a blank as a reference. Cytotoxicity was expressed as a percentage of the negative control (0.1% or 0.2% DMSO).

#### 4.4.3. TPC Assay

The assay was based on staining total cellular protein [14]. After the incubation, the medium containing the drug was removed and 100 µL of coomassie brilliant blue R-250 dye was added to each well. The plate was shaken for 10 min. Then the stain was removed and the cells were rinsed twice with 100 µL of washing solution (glacial acetic acid/ethanol/water, 5:10:85, *v*:*v*:*v*). After that, 100 µL of the desorbing solution (1 M potassium acetate) was added and plates were shaken again for 10 min. The absorbance was measured at 595 nm in a microplate reader using blank as a reference. Cytotoxicity was expressed as a percentage of the negative control (0.1% or 0.2% DMSO).

#### 4.4.4. LDH Leakage Assay

The integrity of the plasma membrane was assessed through the test of lactate dehydrogenase (LDH) release, which was monitored using a commercially available Cytotoxicity Detection Kit (LDH) (Roche Diagnostics, Basel, Switzerland) [15]. The medium (100 µL/well) without cells was transferred into the corresponding wells of an optically clear 96-well flat bottom microplate and a 100-µL reaction mixture was added to each well. Then the plates were incubated for 30 min at room temperature in darkness. After that time, 50 µL/well of 1 M HCl was added to stop the reaction. The absorbance was measured at 492 nm in a microplate reader using a blank as a reference.

### 4.5. Analysis of Drug Interactions

The nature of the interaction between SAL and tiamulin or prednisolone was analyzed using the combination index (CI) introduced by Chou and Talalay for quantifying synergism or antagonism of two drugs [35]. The tested concentrations of both tiamulin (1 or 10 µg/mL) and prednisolone (1 µg/mL) were previously proven to be non-cytotoxic for all tested cell models [11]. The CI (combination index) was calculated, which is a mathematically compiled algorithm for the pharmacological interaction of two drugs and denominates its nature. CI = (C)1/(x)1 + (C)2/(x)2, where (C)1 or (C)2 represents the concentrations of drugs 1 or 2 used in combination to achieve x% cell viability after exposure to drug 1 ((x)1) or drug 2 ((x)2). Values of CI ≤ 0.9, between >0.9 and <1.1 and ≥1.1 indicate synergism, additive effect, and antagonism, respectively.

### 4.6. Determination of SAL Metabolites

#### 4.6.1. Sample Preparation

The medium samples from microplate wells were collected for identifying SAL metabolites and normalized to a cell number. An aliquot of 200 µL of each sample was measured to Eppendorf tube. Next, 100 µL of 40% sodium acetate solution and 500 µL of acetonitrile were added. The samples were vortex-mixed for 30 s and centrifuged (14 000 rpm, 5 min). The upper organic phase was transferred to a clean glass tube and evaporated to dryness under the stream of nitrogen at a temperature of 45 °C. The dry residue was reconstituted in 50% acetonitrile and analyzed with LC-MS/MS.

#### 4.6.2. LC-MS/MS Determination

The analysis was performed on 1260 (Agilent, Palo Alto, CA, USA) coupled with 5500 QTrap (Sciex, Framingham, MA, USA). The system was operated by Analyst 1.6.2 software. SAL metabolites were separated on Poroshell 120 EC-C18, 2.1 mm × 100 mm, 2.7 μm column (Agilent, Palo Alto, CA, USA) coupled with pre-column. Gradient elution was applied with acetonitrile: methanol mixture (60:40, *v*:*v*) used as phase A and 0.01 ammonium formate pH 4.0 (phase B). The gradient was as follows: 0–1 min 20% A, 1.5–14 min 95%, from 14.5 min re-equilibration with 20% A. The column temperature was kept at 55 °C, the flow of the mobile phase was 250 µL/min, and the injected volume was 10 µL.

The detection was performed in an electrospray positive ionization mode (ESI+). The parameters of an ion source and collision cell were optimized for SAL and were as follows: the source temperature 475 °C, capillary voltage +5.5 kV, curtain gas 20 psi, both nebulizer gas and turbo heater gas 20 psi, de-clustering potential 6 eV, entrance potential 10 eV, collision energy 65 eV, and collision exit potential 20 eV. The collision gas was set as high.

SAL (*m*/*z* 773.4 to *m*/*z* 431.3) was monitored in multiple reaction monitoring (MRM) modes. Based on theoretical biotransformation of SAL, 55 MRM transitions were designed and included in the method. Additionally, information dependent analysis experiments were performed in a full scan mode (enhanced product ion spectra of four most abundant MRM transitions).

#### 4.6.3. Data Analysis

The signals for further analysis were selected with the LightSight 2.3 software. Extracts from control samples (media incubated with corresponding concentrations of SAL) were used as reference chromatograms. Based on this analysis, seven MRM transitions were chosen as containing potential SAL metabolites. These MRM transitions (with 20 chromatographic peaks) were then quantified with Multiquant 2.1. software. Quantitative analysis was performed for SAL. For the metabolites, the ratio of the signals was used as a semi-quantitative parameter because no reference standards are available.

### 4.7. Statistical Analysis

The results of the tests were expressed as mean ± SD (standard deviation). The experiments were performed in three independent repetitions (*n* = 3). Statistical analysis was performed using Prism5 (Version 5.0, GraphPad, San Diego, CA, USA). One way analysis of variance (ANOVA) followed by Dunnett’s post-hoc test was applied. The values indicating cytotoxicity concentration (EC_50_) at two time points were calculated by GraphPad Prism 5.0 and expressed as mean ± SEM (standard error of mean). Statistical evaluation was performed using ANOVA, which was followed by Tukey’s post-hoc test. Differences with *p* ≤ 0.05 were considered statistically significant.

## Figures and Tables

**Table 1:** The effective concentrations (EC_50,_ µg/mL) determined in primary human hepatocytes (PHH), HepG2 and Balb/c 3T3 cells by MTT, NRU, TPC, and LDH assays after 12, 24, and 48 h exposure to salinomycin (SAL) and its combination with tiamulin at concentrations 1 µg/mL (SAL + T1) and 10 µg/mL (SAL + T10) or prednisolone at concentration 1 µg/mL (SAL + P). Data are presented as mean ± SEM (*n* = 3, independent experiments).

Cell Model	Assay	Time	SAL	SAL + T1	SAL + T10	SAL + P
PHH	MTT	12 h	>25	>25	N/A*	>25
		24 h	>25	>25	N/A	>25
	NRU	12 h	20.4 ± 1.9 ^a^	4.4 ± 2.3 ^b^	N/A	8.2 ± 2.5 ^c^
		24 h	19.8 ± 1.6 ^a^	2.2 ± 0.6 ^b^	N/A	5.2 ± 1.2 ^c^
	TPC	12 h	>25	>25	N/A	>25
		24 h	>25	>25	N/A	>25
	LDH	12 h	12.9 ± 3.2 ^a^	8.7 ± 1.3 ^b^	N/A	12.5 ± 0.7 ^a^
		24 h	11.3 ± 0.7 ^a^	5.9 ± 2.4 ^b^	N/A	11.5 ± 2.2 ^a^
HepG2	MTT	24 h	15.4 ± 1.7 ^a^	13.5 ± 1.1 ^a^	11.0 ± 1.9 ^b^	>25
		48 h	12.9 ± 1.4 ^abc^	12.8 ± 0.2 ^a^	10.5 ± 1.7 ^b^	14.9 ± 1.5 ^c^
	NRU	24 h	0.6 ± 0.2 ^a^	<0.39	<0.39	0.7 ± 0.1 ^a^
		48 h	<0.39	<0.39	<0.39	<0.39
	TPC	24 h	13.3 ± 2.5 ^a^	12.6 ± 1.7 ^a^	4.6 ± 0.6 ^b^	10.5 ± 1.5 ^a^
		48 h	11.3 ± 1.3 ^a^	9.6 ± 0.7 ^a^	3.0 ± 0.1 ^b^	4.9 ± 1.2 ^c^
	LDH	24 h	>25	18.5 ± 1.1 ^b^	18.4 ± 1.1 ^b^	>25
		48 h	13.8 ± 0.2 ^a^	12.1 ± 1.3 ^ac^	5.6 ± 0.4 ^b^	11.4 ± 1.1 ^c^
Balb/c 3T3	MTT	24 h	10.4 ± 1.4 ^a^	4.5 ± 0.7 ^b^	4.9 ± 0.7 ^b^	16.4 ± 1.5 ^c^
		48 h	4.3 ± 0.9 ^a^	3.7 ± 0.7 ^ac^	2.3 ± 0.4 ^b^	2.6 ± 0.5 ^bc^
	NRU	24 h	0.6 ± 0.2	<0.39	<0.39	<0.39
		48 h	<0.39	<0.39	<0.39	<0.39
	TPC	24 h	7.9 ± 1.1 ^a^	3.9 ± 0.7 ^b^	3.1 ± 0.7 ^b^	12.3 ± 2.0 ^c^
		48 h	1.4 ± 0.5 ^ac^	0.9 ± 0.2 ^a^	0.5 ± 0.1 ^b^	1.8 ± 0.3 ^c^
	LDH	24 h	>25	>25	>25	>25
		48 h	23.1 ± 0.8 ^a^	22.6 ± 1.5 ^a^	19.1 ± 1.6 ^a^	24.8 ± 1.2 ^a^

* N/A—not analysed. The different small letters (a–c) within lines indicate significant differences between the treatments at *p* ≤ 0.05.

**Table 2:** Potential metabolites of salinomycin identified in the medium of the primary human hepatocytes (PHH).

ID	Relative RT ^a^	Biotransformation Route	Ratio to Control ^b^	% of Signal of All Metabolites in PHH			Detected In		
				SAL	SAL + P	SAL + T	HepG2	Balb/c 3T3	
M1	0.501	Trihydroxylation	1424	1.64	1.40	0.61			
M2	0.552	Dihydroxylation	5357	4.00	4.00	2.59	Yes, P ^c^		
M3	0.563	Dihydroxylation	468	1.09	1.05	0.59			
M4	0.573	Dihydroxylation	414	0.83	0.40	0.42			
M5	0.617	Dihydroxylation	∞ ^d^	0.20	0.20	0.13			
M6	0.621	Dihydroxylation	6167	15.36	14.18	9.59	Yes, P		
M7	0.644	Dihydroxylation	∞	0.27	0.25	0.16			
M8	0.656	Dihydroxylation	408	1.08	1.12	0.97			
M9	0.670	Dihydroxylation	490	1.10	1.10	1.02			
M10	0.682	Hydroxylation + Demethylathion	∞	0.05	0.05	0.05			
M11	0.691	Demethylathion	11.3	0.04	0.04	0.03			
M12	0.703	Hydroxylation	190	48.1	49.9	52.0	Yes		
M13	0.709	Dihydroxylation	155	0.22	0.22	0.31			
M14	0.722	Hydroxylation	∞	23.9	23.6	28.4	Yes		
M15	0.733	Dihydroxylation	155	0.17	0.18	0.28			
M16	0.789	Dihydroxylation	29.6	0.19	0.22	0.24			
M17	0.801	Hydroxylation	59.4	1.33	1.39	1.86			
M18	0.851	Demethylathion	48.5	0.04	0.22	0.32			
M19	0.866	Dihydroxylation	14.0	0.29	0.33	0.28			
M20	0.956	Dihydroxylation	3.00	0.12	0.13	0.12			

^a^ Relative RT in comparison to SAL. ^b^ Mean ratio of metabolite peak areas in PHH samples to the signal of respective controls (medium samples incubated without cells). Metabolite IDs were assigned to the signals that gave such a ratio of at least 2. ^c^ P—found only in prednisolone co-treated samples. ^d^ ∞—no signal was found in control samples.

## Supplementary Materials

The following are available online, Figure S1: The cytotoxicity of salinomycin (SAL) and its combination with tiamulin (T) or prednisolone (P) at concentration 1 µg/mL after 12 and 24 h exposure of PHH. Results were calculated as the percent of solvent control (mean ± SD) (*n* = 3, independent experiments). Statistical significance was evaluated by ANOVA and Dunnet’s post-test * *p* ≤ 0.05. # *p*≤ 0.01 and *p* ≤ 0.001. Triton X was a positive control. Figure S2: The cytotoxicity of salinomycin (SAL) and its combination with tiamulin at concentration 1µg/mL (T1) and 10 µg/mL (T10) or prednisolone at concentration 1 µg/mL (P1) after 24 and 48 h exposure of HepG2 cells. Results were calculated as the percentage of solvent control (mean ± SD) (*n* = 3, independent experiments). Statistical significance was evaluated by ANOVA and Dunnet’s post-test * *p* ≤ 0.05, # *p* ≤ 0.01, and *p* ≤ 0.001. Triton X was a positive control. Figure S3: The cytotoxicity of salinomycin (SAL) and its combination with tiamulin at concentration 1µg/mL (T1) and 10 µg/mL (T10) or prednisolone at concentration 1 µg/mL (P1) after 24 and 48 h exposure of fibroblasts (Balb/c 3T3). Results were calculated as the percentage of solvent control (mean ± SD) (*n* = 3, independent experiments). Statistical significance was evaluated by ANOVA and Dunnet’s post-test * *p* ≤ 0.05, # *p* ≤ 0.01, and *p* ≤ 0.001. Triton X was a positive control. Figure S4: Selected Reaction Monitoring chromatograms of potential salinomycin metabolites in medium of PHH exposed to salinomycin at the concentration of 25 μg/mL for 24 h.
