# Supplementary material for: Primary Human Hepatocytes, But not HepG2 or Balb/c 3T3 Cells, Efficiently Metabolize Salinomycin and Are Resistant to Its Cytotoxicity
Source: Molecules. 2020 Mar 5;25(5):1174. doi: 10.3390/molecules25051174 (PMC7179450; doi:10.3390/molecules25051174)
Supplement: Supplementary file 1 [file molecules-25-01174-s001.pdf]

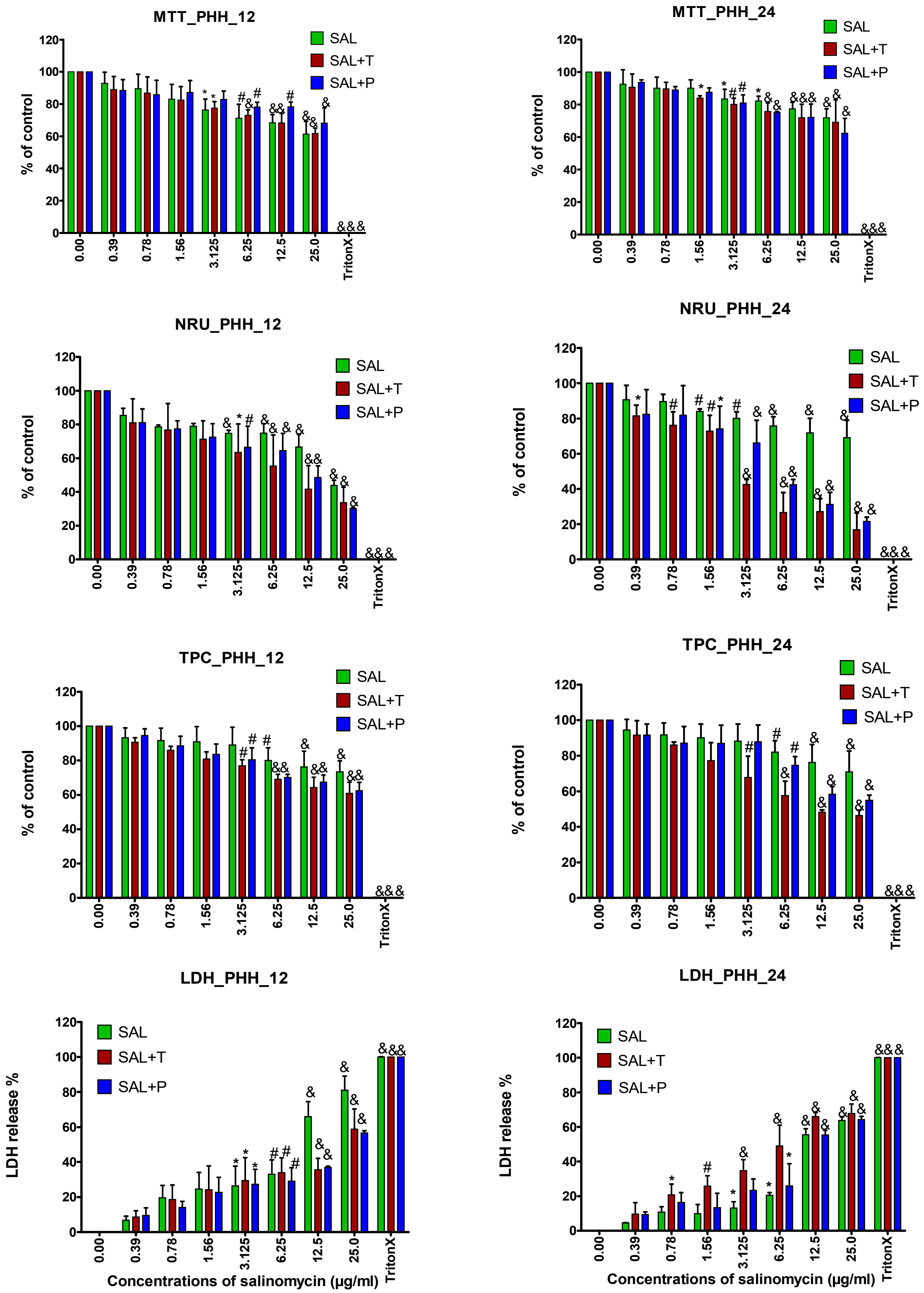

Supplement Figure S1. The cytotoxicity of salinomycin (SAL) and its combination with tiamulin (T) or prednisolone (P) at concentration 1 µg/ml after 12 and 24 h exposition of primary human hepatocytes (PHH). Results were calculated as % of solvent control (mean±SD) (n=3, independent experiments). Statistical significance was evaluated by ANOVA and Dunnet`s post-test *P≤0.05; # P≤0.01; & P≤0.001. Triton X was a positive control.

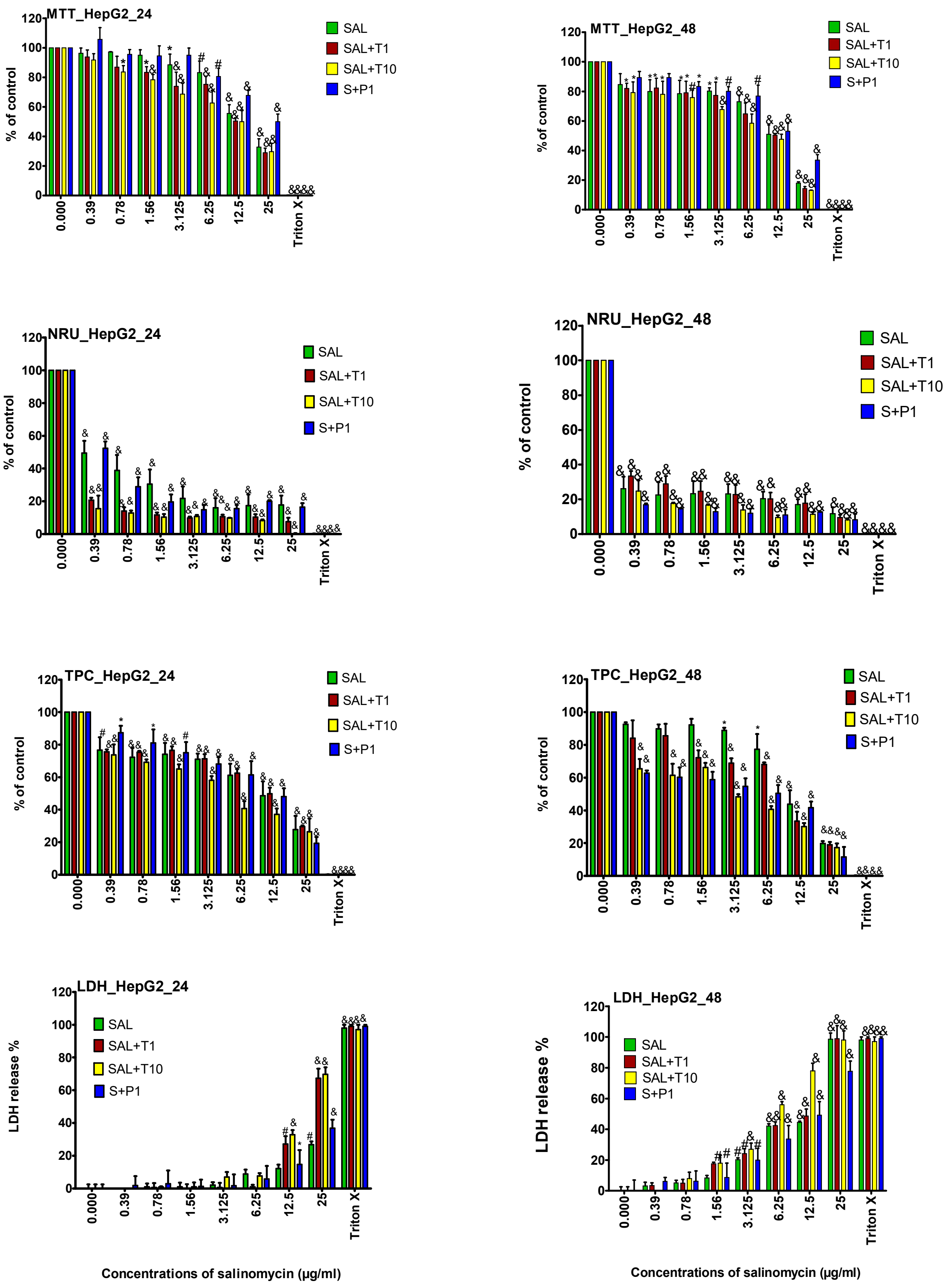

Supplement Figure S2. The cytotoxicity of salinomycin (SAL) and its combination with tiamulin at concentration 1µg/ml (T1) and 10 µg/ml (T10) or prednisolone at concentration 1 µg/ml (P1) after 24 and 48 h exposition of HepG2 cells. Results were calculated as % of solvent control (mean±SD) (n=3, independent experiments). Statistical significance was evaluated by ANOVA and Dunnet`s post-test*P≤0.05; # P≤0.01; & P≤0.001. Triton X was a positive control.

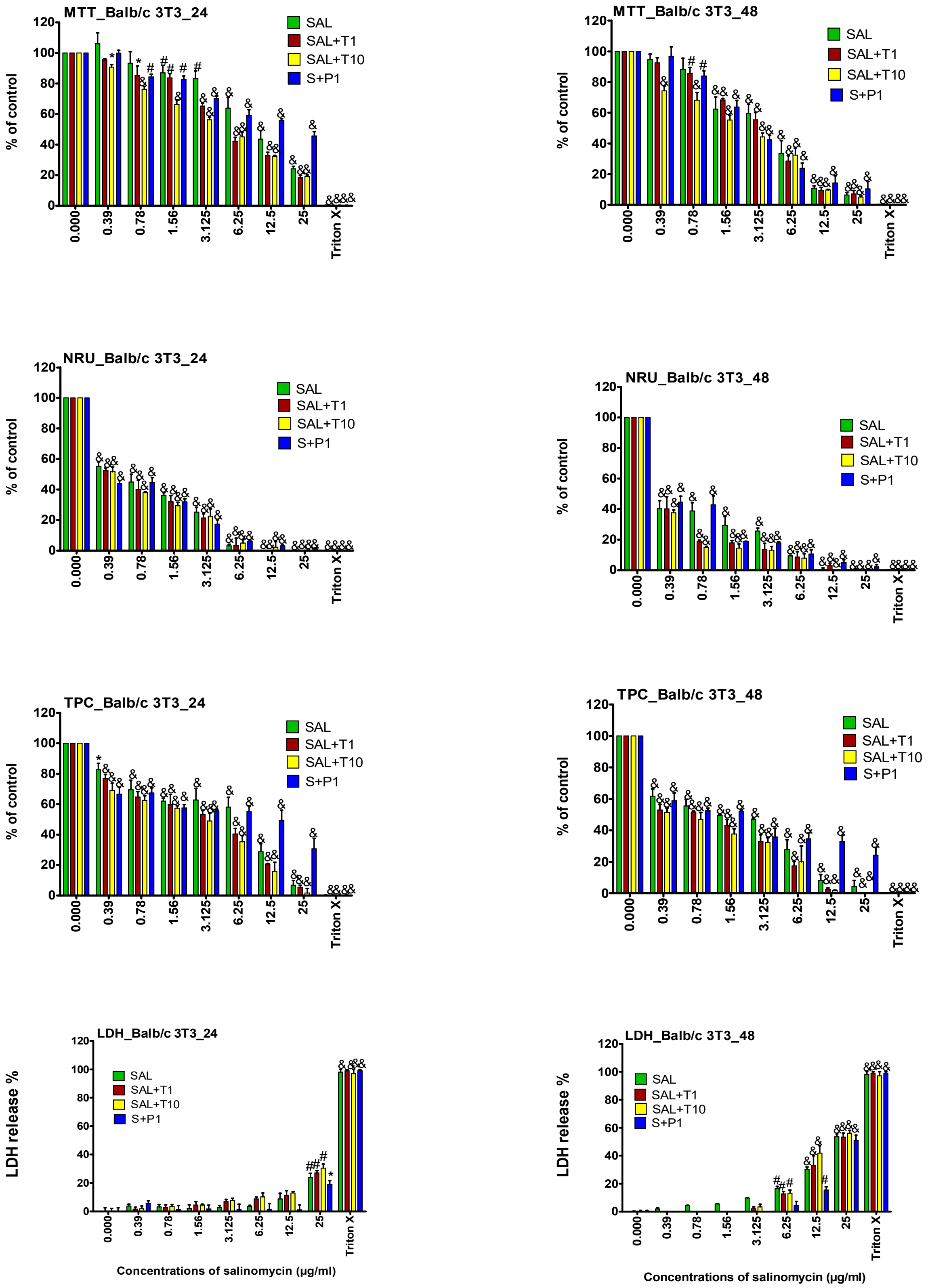

Supplement Figure S3. The cytotoxicity of salinomycin (SAL) and its combination with tiamulin at concentration 1µg/ml (T1) and 10 µg/ml (T10) or prednisolone at concentration 1 µg/ml (P1) after 24 and 48 h exposition of fibroblasts (Balb/c 3T3). Results were calculated as % of solvent control (mean±SD) (n=3, independent experiments). Statistical significance was evaluated by ANOVA and Dunnet`s post-test*P≤0.05; # P≤0.01; & P≤0.001. Triton X was a positive control.

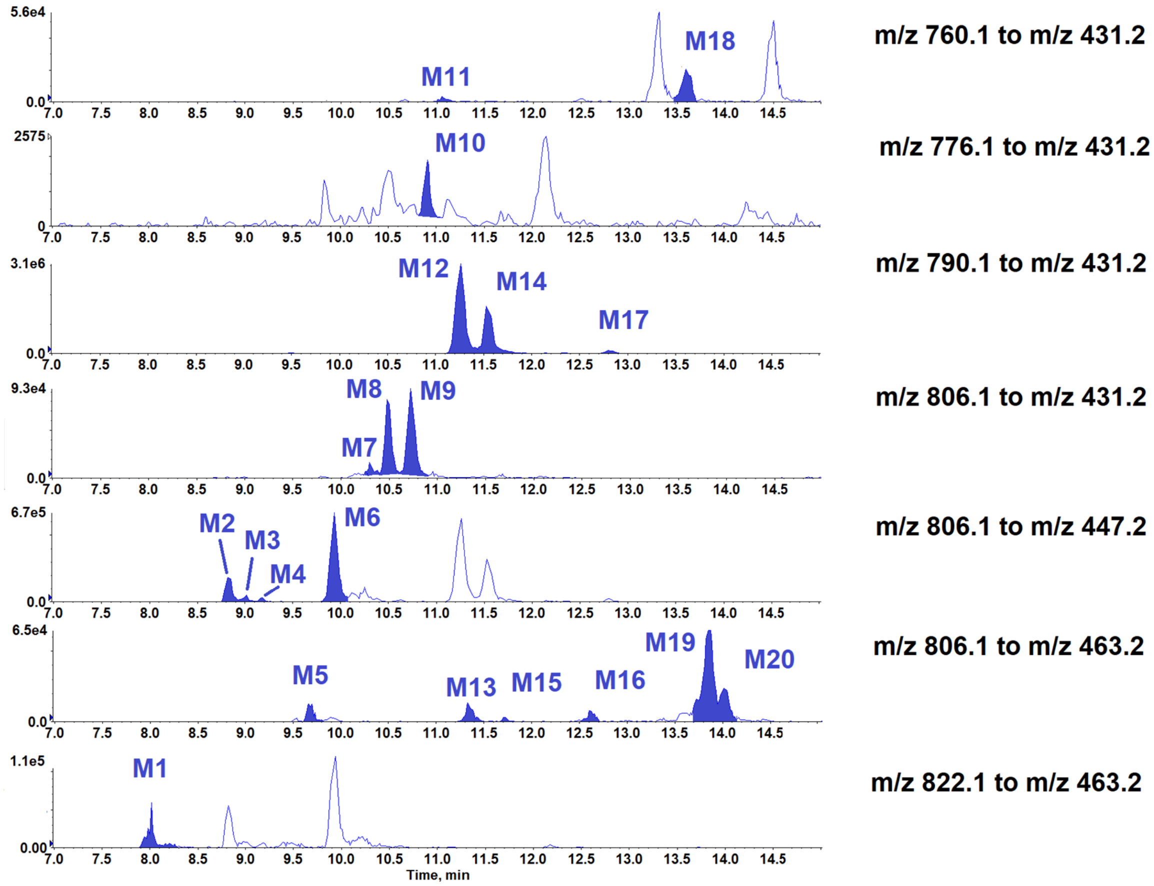

Supplement Figure S4. Selected Reaction Monitoring chromatograms of potential salinomycin metabolites in medium of primary human hepatocytes exposed to salinomycin at the concentration of 25 µg/ml for 24 h.
